# Effect of Alignment on Enhancement of Thermal Conductivity of Polyethylene–Graphene Nanocomposites and Comparison with Effective Medium Theory

**DOI:** 10.3390/nano10071291

**Published:** 2020-06-30

**Authors:** Fatema Tarannum, Rajmohan Muthaiah, Roshan Sameer Annam, Tingting Gu, Jivtesh Garg

**Affiliations:** School of Aerospace and Mechanical Engineering, University of Oklahoma, Norman, OK 73019, USA; fatema.tarannum-1@ou.edu (F.T.); rajumenr@ou.edu (R.M.); anna0003@ou.edu (R.S.A.); Tingting.Gu-1@ou.edu (T.G.)

**Keywords:** thermal conductivity, strain, alignment effect, polymers, graphene, nanocomposites

## Abstract

Thermal conductivity (*k*) of polymers is usually limited to low values of ~0.5 Wm^−1^K^−1^ in comparison to metals (>20 Wm^−1^K^−1^). (100)_T3_//(926)_T4_ The goal of this work is to enhance thermal conductivity (*k*) of polyethylene–graphene nanocomposites through simultaneous alignment of polyethylene (PE) lamellae and graphene nanoplatelets (GnP). Alignment is achieved through the application of strain. Measured values are compared with predictions from effective medium theory. A twin conical screw micro compounder is used to prepare polyethylene–graphene nanoplatelet (PE-GnP) composites. Enhancement in *k* value is studied for two different compositions with GnP content of 9 wt% and 13 wt% and for applied strains ranging from 0% to 300%. Aligned PE-GnP composites with 13 wt% GnP displays ~1000% enhancement in *k* at an applied strain of 300%, relative to *k* of pristine unstrained polymer. Laser Scanning Confocal Microscopy (LSCM) is used to quantitatively characterize the alignment of GnP flakes in strained composites; this measured orientation is used as an input for effective medium predictions. These results have important implications for thermal management applications.

## 1. Introduction:

Miniaturization accompanied with integration of additional functionalities in electronic devices has resulted in a continuous increase in power dissipation. Efficient heat dissipation in these devices is crucial to maintain chip temperatures below permissible levels allowing optimum performance and reliable service life [1,2]. Lightweight polymer composites with enhanced thermal conductivity can enable dissipating required levels of heat fluxes. The goal of this research is to enhance thermal conductivity of polyethylene using graphene nanoplatelets (GnPs) as a filler by simultaneously aligning polyethylene lamellae and GnPs along the direction of heat transfer. 

Polymers are used in a wide spectrum of applications ranging from electronic packaging to aerospace materials. Because of their unique advantages such as light weight, Fig. good chemical resistance, good corrosion resistance, and excellent processability, polymers provide avenues to replace metals in thermal management applications [3,4,5,6]. Polymers, however, typically have lower thermal conductivity (<0.5 W$m^{–1}K^{–1}$) compared to metals (>20 W$m^{–1}K^{–1}$), which limits their application in thermal management. The low thermal conductivity of polymers is in part due to the random orientation of polymer lamellae (crystalline regions) and presence of amorphous regions in between crystalline regions. Entanglement and impurities present in those regions lead to an increase in phonon scattering which diminishes thermal transport [7]. Studies have shown that orienting polymer chains along one direction can lead to significant enhancement in *k* [7,8,9,10,11]. Several methods for inducing polymer chain orientation have been investigated, which include deformation by simple shear [11], mechanical strain [12], gel spinning [13,14,15] and super drawing [16,17,18]. Singh et al. achieved 20-fold enhancement in *k* value (4.4 W$m^{–1}K^{–1})\;$of a vertically aligned array of polythiophene fibers by electropolymerization, in comparison to bulk polymer, due to aligned chain orientation [19]. The *k* of a single PE fiber with ultra-aligned PE chains was measured to be 104 W$m^{–1}K^{–1},\;$almost 200 times larger than the *k* of bulk PE (∼0.5 W$m^{–1}K^{–1}$) [20]. In another report, Ghasemi et al. found that alignment of polymer chains resulted in a thermal conductivity of ∼16 W$m^{–1}K^{–1}$ in polyethylene films [21], at draw ratios approaching ∼100. Choy and co-workers [22] investigated both in plane and out-of-plane thermal conductivity of oriented high-density polyethylene and found the in-plane *k* value to increase to 14 W$m^{–1}K^{–1}$ at 300K for an applied strain of 2500%.

Graphene has emerged as a superior nanofiller compared to carbon nanotubes for enhancing thermal conductivity of polymer composites, owing to its lower thermal boundary resistance (TBR) [23] with polymers in comparison to carbon nanotubes (CNTs). The estimated range of TBR between CNTs and the polymer matrix is $10^{–8}$–$10^{–7}m^{2}$K $W^{–1\;}$[24], whereas the TBR of GnPs is in the range of ~$10^{–9}m^{2}$K $W^{–1}$ [23]. This lower TBR allows efficient heat transfer between polymer and graphene, leading to overall higher thermal conductivity. 

While graphene has very high in-plane thermal conductivity (~1500–5000 W$m^{–1}K^{–1}$) [25,26], its out-of-plane *k* value is low ∼10–20 W$m^{–1}K^{–1\;}$[27]. A random orientation of GnPs causes significant heat to be partly conducted along the less efficient out-of-plane direction, limiting the contribution of GnPs to the enhancement of *k* along a particular direction. Alignment achieves full advantage of the high in-plane *k* of GnPs in enhancing overall composite *k* along a given direction. Several studies have addressed the alignment of nanofiller. Different approaches like mechanical stretching [28], electric field [29,30,31] and magnetic field [32,33,34] have been used for orientation of nanofillers. Mechanical stretching has been used for alignment of both the polymer matrix [35,36] and dispersed filler material [37,38]. Amy et al. reported an enhancement in *k* by a factor of 18 in epoxy-CNT composites with densely aligned arrays of CNTs [39]. Yan et al. used magnetic field to align magnetic GnP-${\mathrm{Fe}}_{3}O_{4}\;$particles in epoxy composites and found it to enhance the *k* value of epoxy by almost 40% in comparison with randomly oriented GnPs [34]. Song et al. used a self-alignment method and achieved a *k* value of 6.168 W$m^{–1}K^{–1}$ for nano fibrillated cellulose (NFC)/reduced graphene oxide (RGO) nanosheets composite film [40].

Simultaneous alignment of both the polymer lamellae and GnPs along a given direction can result in significant enhancement in thermal conductivity values along that direction. In this study, we explore such simultaneous alignment effect by using mechanical strain; thermal conductivity of PE-GnP composites is measured using the Angstrom method [41]. Laser Scanning Confocal Microscopy [42,43] is used to visualize GnP alignment within composite samples and ImageJ Software (v1.53c) is used to derive quantitative information regarding the average angle of orientation of GnPs with respect to the direction of applied strain for different strains. Characterization of GnP using confocal microscopy is a unique technique to observe the changes in alignment with applied strain, both visually as well as quantitatively. Scanning electron microscopy (SEM), X-ray photoelectron spectroscopy (XPS), Raman spectroscopy and Atomic force microscopy (AFM) are used to characterize GnPs. Finally, measured enhancement in *k* value is compared with predictions of effective medium theory [44].

## 2. Experimental Work

Polyethylene–graphene nanocomposites were prepared using a DSM Xplore micro-compounder (Xplore Instruments, Netherlands) (Figure S1) and mechanically strained using motorized slide (Figure S2). The processes involved in preparing the composites and mechanically straining them are described in the Supplementary Section. Below, we present measurements of thermal conductivity of prepared composites, characterization of GnP alignment using LSCM and effective medium theory used for predictions of thermal conductivity of aligned composites. 

### 2.1. Thermal Conductivity Measurement

**Figure 1:**
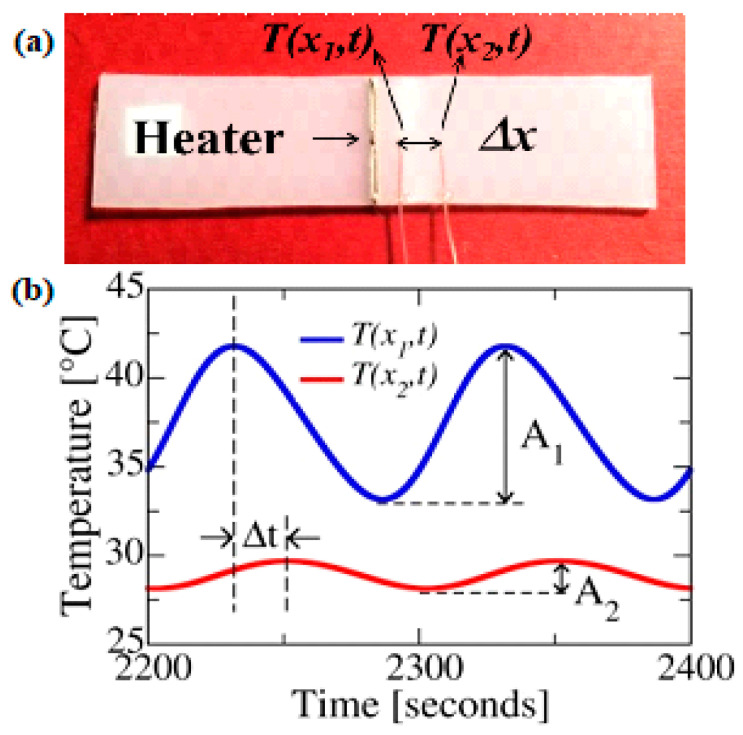
(**a**) Sample prepared for Angstrom method (**b**) Measured temperature response at two different locations on the sample.

Thermal conductivity *k* is obtained from the knowledge of specific heat ($C_{p}$), density (*ρ*), and thermal diffusivity (*α*) using *k* = *αρ*$C_{p}$. Thermal diffusivity is measured in this work using the Angstrom method [41]. The method involves applying a periodic heat pulse in the middle of the sample. For this, a high resistance wire with a resistance of 262.5 Ω/m was embedded in a notch made in the middle of the composite sample and a thermally conductive adhesive paste was used to establish good thermal contact between the wire and the sample (Figure 1a). The wire was used to apply sinusoidal heat signal. Two thermocouples are attached to the sample at distances of 2 and 6 mm from the heater, respectively (Figure 1a). Amplitude of temperature response decays along the length of the sample and simultaneously experiences a phase shift (Figure 1b). Thermal diffusivity is obtained through the knowledge of temperature amplitude and phase shift through the equation, $\alpha =L^{2}/\left(\left[2\times \Delta t\times \ln\left(M/N\right)\right]\right)$, where *L* is the distance between temperature sensors, Δ*t* is the phase difference between two temperature responses, M and N are the temperature amplitudes measured at the two locations. A function generator supplies sinusoidal heat pulse to the heater. The sample is placed inside a high vacuum chamber to eliminate convective heat losses. A turbo pump (Pfeiffer HiCube, USA) is used to evacuate the chamber to pressures down to 10 mtorr. The experimental set-up used to measure the thermal diffusivity is presented in Figure S3. Specific heat and density are measured using Differential Scanning Calorimeter (DSC-Q1000) (TS Instruments, USA) and Pycnometer (AccuPyc 1340V2.0) (Micromeritics, USA) respectively. The accuracy of the measurement set-up was established through good agreement between the measured *k* of the pristine PE (~0.5 W$m^{–1}K^{–1}$) and literature [11].

### 2.2. Characterization of Graphene

**Figure 2:**
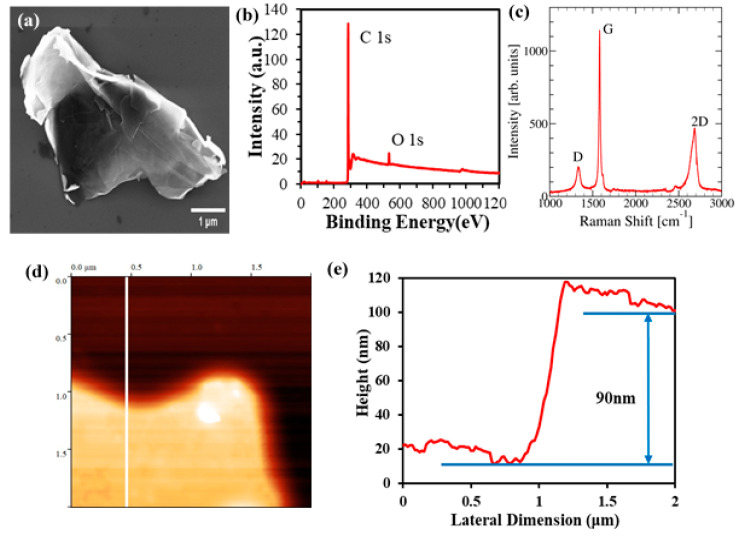
(**a**) SEM image, (**b**) XPS spectrum, (**c**) Raman Spectrum, (**d**) Atomic force microscopy (AFM) image, (**e**) AFM thickness profile.

GnPs (graphene nanoplatelets) were characterized using SEM (JEOL, Boston, Massachusetts, USA), XPS (PHI 5800 X-ray Photoelectron Spectrometer, Physical Electronics, Minnesota, USA), Raman spectroscopy (Horiba, USA) and AFM (Digital Instruments, New York, USA). GnPs used in this work had an average lateral size of ~5 μm and average thickness of 60 nm. Figure 2a shows an SEM image of a GnP with lateral size in the vicinity of ~5 μm. Figure 2d,e show an AFM image of GnP indicating a thickness of 90 nm. X-ray Photoelectron Spectroscopy was used to characterize the chemical composition of GnPs and showed a carbon content of 95.54% along with a minor oxygen content of 3.41%. GnPs were also characterized using Raman spectroscopy as shown in Figure 2c. The low defect density of GnPs used in this work is evident from the low intensity of the D peak and the ratio of D/G peaks.

### 2.3. Confocal Microscopy for Characterization of Orientation of Graphene Nanoplatelets (GnPs) 

To characterize the alignment of GnPs in strained samples, we used Laser Scanning Confocal Microscopy (LSCM). A confocal microscope creates sharp images of a specimen by excluding light from the specimen that is not from the focal plane. The image has less haze and better contrast than that of a conventional microscope and represents a thin cross-section of the specimen. Point-to-point illumination of the specimen and the pinhole apertures are the key features of the modern confocal microscope [45].

**Figure 3:**
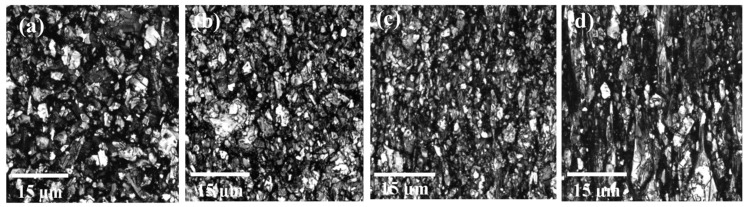
Confocal microscopy images of graphene nanoplatelets (GnP) alignment in strained polyethylene (PE)-GnP composites with strains of (**a**) ε = 0%, (**b**) ε = 100%, (**c**) ε = 200% and (**d**) ε = 300% (Scale bar = 15 μm).

A Leica SP8 laser scanning confocal microscope with a 561 nm DPSS (Diode pumped solid state) laser was used for imaging in this study. The samples were imaged with a 63x/1.4 oil immersion objective with the pinhole aperture at 0.2 AU (Airy unit) and voxel dimensions of 90.2 nm ×90.2 nm × 166.4 nm and to a depth of 11 μm. Figure 3 shows LSCM images of GnPs in PE-GnP (9 wt%) nanocomposite sample, for different strains varying from 0% to 300%. While GnPs are seen to be randomly oriented in Figure 3a, Figure 3b–d clearly show alignment of GnPs along the stretch direction (vertical direction shown in these figures). As a next step, we processed these images using ImageJ software to extract quantitative understanding of orientation of GnP in strained samples.

### 2.4. Angle Measurement Between GnP and Draw Direction

**Figure 4:**
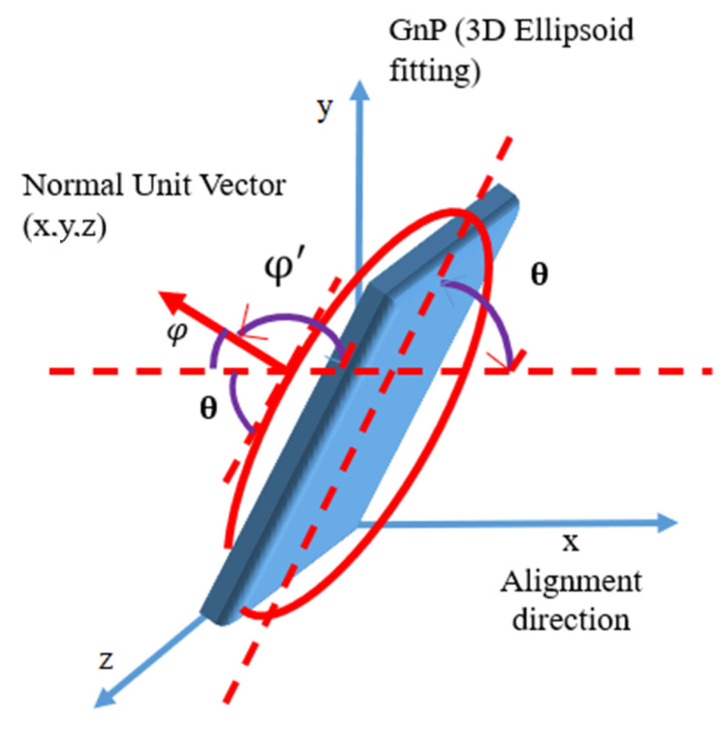
Calculation of the angle between GnP and stretching direction.

Using confocal optical sectioning, an image of a section is captured from the PE-GnP composite sample. The section is in the x-y plane with thickness along the z direction. Stacking such sections creates a 3D image of the sample. From the 3D image, average angle between GnP and stretching direction can be measured using ImageJ software v1.53c. To find out the angle, all slices within a certain thickness are used for detecting GnP particles in each image. Using the 3D object counter tool in ImageJ software, the 3D particles were detected. Then, the 3D ellipsoid fitting tool within the software is used to fit ellipsoids to individual particles. The tool provides detailed quantitative information for each ellipsoid, including its orientation. Specifically, the coordinates of three main elongation axis of 3D ellipsoids are provided. The vector of the shortest elongation axis is considered as a normal vector to the ellipsoid and provides a way to quantify orientation of the ellipsoid, as shown in Figure 4. The direction angle of the normal vector with respect to the stretch direction is calculated using the formula, $\varphi =\cos^{-1}\left[x/\sqrt{x^{2}+y^{2}+z^{2}}\right]$, where x is the direction of applied strain. The angle between the graphene nanoplatelet and alignment direction is θ = 90° – φ. The average angle is measured by calculating angles of orientation for approximately 3000–4000 GnPs for each sample. Analysis was repeated for different locations within a sample and for samples with different weight percentages of GnPs and for different strains varying from 0% to 300%.

### 2.5. Effective Medium Theory (EMT)

The measured effect of alignment on the enhancement of thermal conductivity is also compared against predictions of effective medium theory. We use the effective medium theory presented by Nan et al. [44] as it can account for both orientation of the dispersed phase as well as interfacial thermal resistance between dispersed particles and polymer matrix. According to the theory, the effective thermal conductivity of aligned PE-GnP composite can be predicted by using the following equation,

(1) $$\;k_{effective}=k_{m}\frac{2+f\left[\beta_{11}\left(1-L_{11}\right)\left(1+\langle \cos^{2}\theta \rangle \right)+\beta_{33}\left(1-L_{33}\right)\left(1-\langle \cos^{2}\theta \rangle \right)\right]}{2+f\left[\beta_{11}L_{11}\left(1+\langle \cos^{2}\theta \rangle \right)+\beta_{33}L_{33}\left(1-\langle \cos^{2}\theta \rangle \right)\right]}$$

where $k_{m}$ is the thermal conductivity of pristine polymer and $k_{effective}$ is the effective thermal conductivity of polymer–graphene nanocomposite with a volume fraction, f, of GnPs. The orientation of GnPs is taken into account in the above equation through the parameter $\langle \cos^{2}\theta \rangle$, where θ is the angle between GnPs and the draw direction, and $\langle \cos^{2}\theta \rangle$ is an ensemble averaged value of $\cos^{2}\theta$ over all GnPs ($\langle \cos^{2}\theta \rangle =1/3\;$ for a completely random orientation and $\langle \cos^{2}\theta \rangle =1$ for perfectly aligned GnPs). 

$L_{ii}$ are geometrical parameters and depend upon the aspect ratio (*p*) of graphene nanoparticle (aspect ratio is the ratio of the thickness, t, to the lateral dimension, L, of the nanoplatelets). For oblate inclusions such as nanoplatelets, where p = t/L < 1, these geometrical parameters,$\;L_{ii}$, are computed using the following equations

(2) $$\;L_{11}=L_{22}=\frac{p^{2}}{2\left(p^{2}-1\right)}\;+\frac{p}{2{\left(1-p^{2}\right)}^{3/2}}\cos^{-1}p$$

(3) $$L_{33}=1\;-\;2L_{11}$$

The parameters $\beta_{ii}$ in Equation (1) above are computed using,

(4) $$\beta_{ii}=\frac{K_{ii}^{c}-k_{m}}{k_{m}+L_{ii}\left(K_{ii}^{c}-k_{m}\right)}$$

where, $K_{ii}^{c}$ are the effective values of nanoplatelet thermal conductivity along different cartesian directions, based on including the effect of interface thermal resistance. The effective thermal conductivities of the nanoplatelet along in-plane ($K_{11}^{c}$ and $K_{22}^{c}$) and out-of-plane ($K_{33}^{c}$) are, respectively,

(5) $$K_{11}^{c}=K_{22}^{c}=\frac{k_{in}}{1+\gamma L_{11}k_{in}/k_{m}}$$

(6) $$K_{33}^{c}=\frac{k_{out}}{1+\gamma L_{33}k_{out}/k_{m}}$$

where, γ=(1+2p)α and α ($=Rk_{m}/t$) is a dimensionless parameter related to interface thermal resistance, R. $k_{in}$ and $k_{out}$ represent the in-plane and out-of-plane thermal conductivity of graphene nanoplatelet (GnP), respectively.

## 3. Result and Discussion

**Figure 5:**
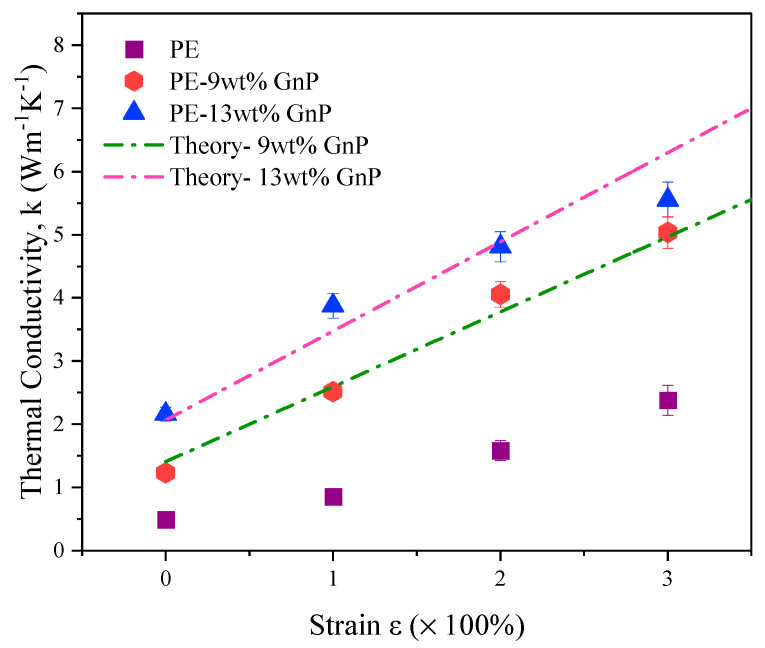
Thermal conductivity enhancement of pure PE, PE/GnP (9 wt%) and PE/GnP (13 wt%) as a function of strain.

Measured thermal conductivity values of PE-GnP composites with 9 wt% and 13 wt% GnP content with strains between 0% to 300% are displayed in Figure 5. Thermal conductivity values for pure PE are also presented for comparison. 

The thermal conductivity for unoriented pure PE is measured to be ∼0.5 W$m^{-1}K^{-1}$. For the oriented sample with an applied strain of 300%, the measured thermal conductivity of the PE sample reaches *k* = 2.3 W$m^{-1}K^{-1}$. This increase in thermal conductivity in strained PE sample is due to the alignment of polymer lamellae. Figure 5 further shows that thermal conductivity of PE-GnP composites is higher than pristine PE samples, indicating the beneficial effect of adding GnPs. For 9 wt% GnP composite, *k* values of 1.23 W$m^{-1}K^{-1}$ and 5.03 W$m^{-1}K^{-1}$ are achieved for 0% and 300% strain, respectively. A higher amount of filler content in the polymer matrix enhances the thermal conductivity value even further. Further, the rate of increase of thermal conductivity with respect to applied strain is higher for PE-GnP composites compared to pristine polymer samples. This higher slope is due to the additional beneficial effect of alignment of GnPs within the strained PE-GnP composites, while in pristine PE samples, only the alignment of polymer lamellae contributes to thermal conductivity enhancement. This slope is observed to increase with an increase in GnP content, further indicating that alignment of larger amount of GnPs leads to greater increase in thermal conductivity. 

The above measurements clearly demonstrate the beneficial effect of simultaneous alignment of polymer and graphene. Figure 5 also shows a good agreement between measurements and predictions of the effective medium theory. 

In Table 1, we also report thermal conductivity enhancements in aligned systems reported in other recent works. The measured thermal conductivity enhancement in our work is seen to be comparable to the values in Table 1. 

To achieve prediction through effective medium theory, we derived alignment of nanoplatelets using confocal microscopy. While the effect of strain on the alignment of polymer lamellae has been quantified in our earlier works through the use of wide-angle X-ray scattering (WAXS) [43], this work is the first to quantify alignment of GnPs in strained samples using confocal microscopy. For 9 wt% PE-GnP nanocomposite, the average angle of GnPs with respect to draw direction was measured to decrease from 39° for 0% strain to 25.7° for 300% (Table 2). 

Analysis for the 13 wt% sample showed a similar decrease in GnP angle from 41.3° for zero strain to 25.0° for the sample with 300% applied strain. Clearly, as the applied strain is increased, the average angle between GnP and stretching direction decreases, indicating progressive alignment of GnPs.

These angles were used as an input for effective medium theory. For *k* prediction, lateral dimension (*L*) and thickness (*t*) of GnPs were taken to be 5 μm and 60 nm, respectively. Interfacial thermal resistance (*R*) is assumed to be 5 $\times 10^{–8}m^{2}K/W$ [24], while in-plane ($k_{in})$ and out-of-plane ($k_{out})$, thermal conductivity of GnPs are taken to be 1000 W$m^{–1}K^{–1}$ and 10 W$m^{–1}K^{–1}$, respectively. Figure 5 shows a reasonable agreement between measurements and predictions based on the above set of parameters. 

A limitation of the effective medium model used in this work, involves the assumption of isotropic thermal conductivity for the base polymer matrix. However, this assumption is true only for the unstrained polymer. For strained polymer, the thermal conductivity along the strained direction is significantly higher compared to other two directions. In this work, the value of base polymer matrix thermal conductivity ($k_{m}$) was taken to be the thermal conductivity along the strained direction. Clearly, this overestimates the average polymer matrix thermal conductivity. The predicted values thus represent an upper bound of the thermal conductivity of aligned composites. 

## 4. Conclusion

In this work, we studied the effect of simultaneous alignment of polyethylene lamellae and graphene nanoplatelets on the thermal conductivity enhancement of PE-GnP nanocomposites. The nanocomposites were fabricated using the melt-mixing method, using a micro-compounder followed by compression molding. Alignment effect was achieved through mechanical stretching of the prepared composites. Laser Scanning Confocal Microscopy (LSCM) was used to quantitatively study the alignment of graphene nanoplatelets. Thermal conductivity of composites along strain direction was measured using the Angstrom method. PE-GnP composites with two different GnP contents of 9 wt% and 13 wt% were studied. The average angle between GnPs and strain direction was measured to decrease from 39° for 0% strain to 25.7° for 300% strain for the 9 wt% sample, and from 41° to 25.0° for the 13 wt% sample, for the same increase in strain, indicating progressive alignment of GnPs with increasing strain. The thermal conductivity of nanocomposites with 9 wt% and 13 wt% composition increased from 1.23 W$m^{–1}K^{–1}$ and 2.16 W$m^{–1}K^{–1}$ for the unstrained case to 5.03 W$m^{–1}K^{–1}$ (9 wt%) and 5.55 W$m^{–1}K^{–1}$ (13 wt%), respectively, for an applied strain of 300%, indicating the beneficial effect of GnP alignment. These experimental values were also found to be in good agreement with theoretical prediction based on effective medium theory.

## Figures and Tables

**Table 1:** Enhancement in thermal conductivity of graphene-polymer nanocomposites through alignment effect.

Material	Filler Content	*k (in plane)* $\left(Wm^{-1}K^{-1}\right)$	Method of Alignment	Ref
$\mathrm{GDY}/{\mathrm{PVDF}}^{\;a}$	1 wt%	3.86	Hot press	[46]
$\mathrm{GNS}/{\mathrm{NR}}^{\;b\;}$	5.78 wt%	3.62	Vulcanization pressure	[47]
GnP/${\mathrm{LDPE}\;}^{c\;}$	7.5 wt%	2	Flow induced	[48]
GnP/${\mathrm{PS}\;}^{d\;}$	10 wt%	0.244	Hot press	[49]
${\mathrm{GWF}}^{\;e\;}/$Polyamide	12 wt%	3.73	Layer by layer stacking	[50]
GnP/Epoxy	15 wt%	2.1	Z-pinning	[51]
GNS/${\mathrm{PFA}\;}^{f}\;$	30 wt%	2.39	Hot compression	[52]
${\mathrm{CF}}^{\;g}$/PA6	30 wt%	0.32	Thermal annealing	[53]
$\mathrm{GNP}-{E\;\mathrm{paper}\;}^{h}$/CF	35 wt%	20	Compression	[54]
GnP/PE	13 wt%	5.5	Mechanical Strain	This work

^a^ GDY/PVDV- Graphdiyne/Polyvinylidene fluoride; ^b^ GNS/NR- Graphene Nanosheet/Natural Rubber; ^c^ LDPE- Low Density Polyethylene; ^d^ PS- Polystyrene; ^e^ GWF - Graphene woven fabric; ^f^ PFA- Perfluoroalkoxy; ^g^ CF- Carbon fiber; ^h^ GnP-E paper- Epoxy coated GnP.

**Table 2:** Measured angle of orientation of GnPs for 9 wt% & 13 wt% PE-GnP composite.

Strain	9 wt% PE-GnP		13 wt% PE-GnP	
	Angle (〈θ〉)	$\langle \cos^{2}\theta \rangle$	Angle (〈θ〉)	$\langle \cos^{2}\theta \rangle$
0%	39.03	0.577	41.33	0.542
100%	32.41	0.668	28.00	0.722
200%	26.98	0.740	25.50	0.756
300%	25.68	0.756	25.00	0.751

## Supplementary Materials

The following are available online at https://www.mdpi.com/2079-4991/10/7/1291/s1, Experimental materials and method are described in this section. Figure S1 shows (a) DSM Xplore micro-compounder (b) twin screws used for melt- mixing polymer and graphene within the micro-compounder (c) polyethylene (PE) powder and graphene nanoplatelets (GnP) and (d) prepared PE-GnP composite sample after compression molding. Figure S2 displays (a) Experimental setup for stretching PE-GnP composites (b) Stretched PE-GnP specimen. Figure S3 shows the experimental setup for measurement of thermal conductivity using the Angstrom method.
