# Supplementary material for: Effect of Alignment on Enhancement of Thermal Conductivity of Polyethylene–Graphene Nanocomposites and Comparison with Effective Medium Theory
Source: Nanomaterials (Basel). 2020 Jun 30;10(7):1291. doi: 10.3390/nano10071291 (PMC7407094; doi:10.3390/nano10071291)
Supplement: Supplementary file 1 [file nanomaterials-10-01291-s001.pdf]

Supplementary Materials

Effect of alignment on enhancement of thermal conductivity of polyethylene-graphene nanocomposites and comparison with effective medium theory

Fatema Tarannum, Rajmohan Muthaiah, Roshan Sameer Annam, Tingting Gu, Jivtesh Garg

School of Aerospace and Mechanical Engineering, University of Oklahoma, Norman, Oklahoma 73019, USA

Experimental Work

Materials and preparation

High density polyethylene (HDPE) with a melt index of 2.2 gram per 10 min and with a molecular weight of $M_n \sim$ 10600 was used in this work. Graphene nanoplatelets used for this study have an average thickness of ∼60 nm and lateral size of about ∼5μm. The PE-GNP nanocomposites were prepared by melt-mixing the polymer pellets and graphene nanoplatelets using a DSM Xplore 5 cc micro-compounder. DSM Xplore 5 cc micro-compounder enables good control over the microstructure of the composite through control of parameters such as mixing time, temperature and screw rotation speed. Mixing time, temperature and speed are the main factors that determine dispersion of GNPs in the polymer matrix. Longer mixing time can provide better dispersion but can also damage the nanoplatelets. A mixing time between 40 to 90 minutes was chosen to achieve uniform dispersion as well as prevent damage to GnPs.

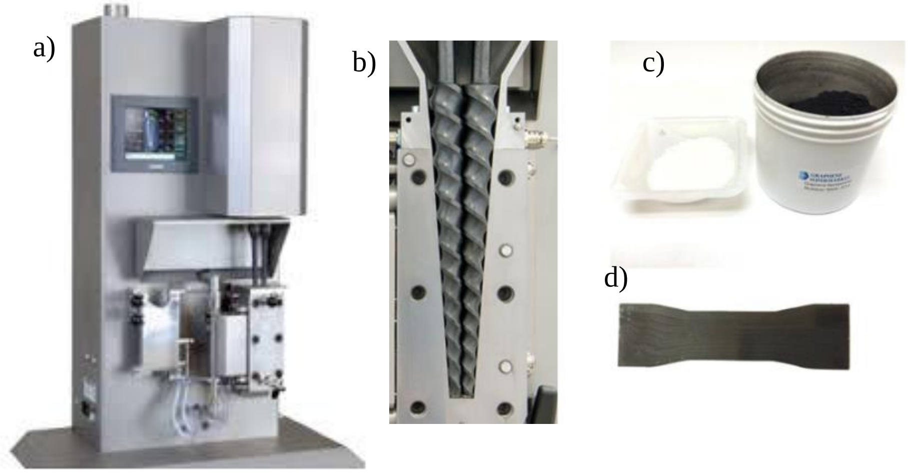

Figure S1: a) DSM Xplore micro-compounder b) twin screws used for melt-mixing polymer and graphene within the micro-compounder c) polyethylene (PE) powder and graphene nanoplatelets (GnP) and d) prepared PE-GnP composite sample after compression molding.

a)
b)
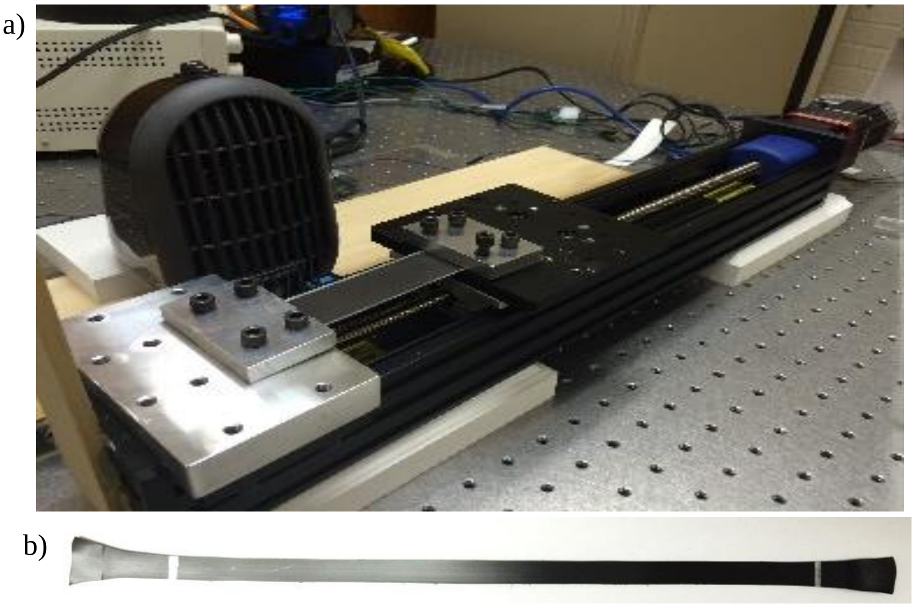

Figure S2: a) Experimental setup for stretching PE-GnP composites b) Stretched PE-GnP specimen.

Similarly, an optimum mixing temperature needs to be chosen to achieve maximum thermal conductivity. High temperature can degrade polymer properties, lowering thermal conductivity. On the other hand, low temperature may prevent uniform dispersion of GNPs due to high melt viscosity. With these considerations, mixing temperature of 160 ℃ was used. Similarly, a rotation speed of 200-220 rpm was chosen in this work. Prepared composites were compression molded to obtain final samples. A pressure of 1 MPa along with a temperature of 145 ℃ was used for 15 min during the compression molding process. The process yielded approximately 1 mm thick specimens. Mechanical strain was applied to the composite samples using a motorized slide (Fig. 2a) to induce the alignment of the graphene nanoplatelets and polymer chains. Low strain rate of ~20 μm/min coupled with heating at 60–70 ℃ was used during the drawing process to prevent brittle failure; the process allowed samples to be strained up to ε =300% (Fig. 2b) (applied strain is represented by $\varepsilon = \Delta l / l_0$, where l_0 is the initial sample length and Δl is the change in length after stretching.

Thermal conductivity measurement set up

The experimental set up for thermal conductivity measurement is shown in Fig. S3. The setup consists of a vacuum chamber connected to a vacuum pump, a data acquisition system, and a function generator. The sample to be measured is placed inside the vacuum chamber, and the function generator supplies the sinusoidal electrical signal to the heater. Data acquisition system is used to measure the temperature response at two locations which provide the thermal diffusivity value.

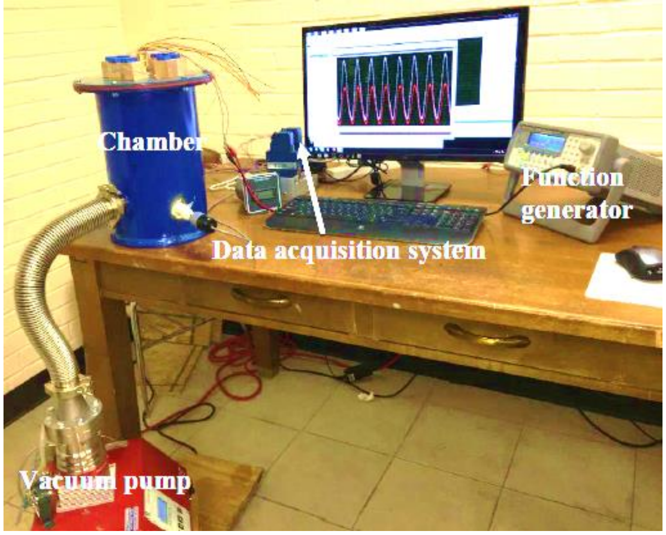

Figure S3: Experimental setup for measurement of thermal conductivity using Angstrom method
